# Emotional Blunting in Hong Kong Patients with Major Depressive Disorder Treated with Vortioxetine: A Naturalistic Observational Study

**DOI:** 10.3390/biomedicines14020270

**Published:** 2026-01-26

**Authors:** Yanni Ip Chi Kwan, C. S. Fung, Sharon K. W. Lee, Vivian W. Y. Lui, Calvin P. W. Cheng

**Affiliations:** Department of Psychiatry, The University of Hong Kong, Pokfulam, Hong Kong; yanni29@hku.hk (Y.I.C.K.); chrisfungcs@connect.hku.hk (C.S.F.); shalee21@hku.hk (S.K.W.L.); wylui221@hku.hk (V.W.Y.L.)

**Keywords:** vortioxetine, emotional blunting, anhedonia, depression, cognitive dysfunction, functional impairment, fatigue, antidepressants

## Abstract

**Background/Objectives**: Major Depressive Disorder (MDD) affects over 280 million people worldwide and is a leading cause of disability. Emotional blunting—characterized by a numbing or flattening of emotions—is a significant yet often underrecognized symptom that impairs daily functioning and interpersonal relationships in patients with MDD. It remains unclear whether emotional blunting results primarily from the disorder itself or from antidepressant treatments, especially selective serotonin reuptake inhibitors (SSRIs) and serotonin–norepinephrine reuptake inhibitors (SNRIs). Vortioxetine, a multimodal antidepressant approved for MDD, may help alleviate emotional blunting by modulating neurotransmitters differently than SSRIs. This study investigates the severity of emotional blunting among Hong Kong MDD patients and explores the changes in this symptom with the use of vortioxetine, while also considering anhedonia as a related dimension of reward processing. **Methods**: This naturalistic, longitudinal observational study in Hong Kong enrolled adults (aged 18 and above) clinically diagnosed with MDD who were initiating vortioxetine treatment for emotional blunting. Patient inclusion was based on independent prescribing decisions by psychiatrists, with informed consent obtained. Data collection comprised one intake interview and the administration of four self-report questionnaires—ODQ, PHQ-9, PDQ-D, SDS, MFI, and SHAPS—at baseline, week 1, week 4, and week 8. Demographic and clinical history data were also recorded. Questionnaires were completed online or via phone, over a study duration of approximately two months. **Results**: The prevalence of emotional blunting, estimated by the proportion of patients with an ODQ score at or above the clinical cut-off (≥50), was 91.9% at baseline, decreasing to 85.5% at week 1, 77.7% at week 4, and 73.3% at week 8. Significant improvements were also observed in depressive symptoms, cognitive dysfunction, functional impairment, pleasure experience, and fatigue. **Conclusions**: In this naturalistic observational cohort of patients with MDD who were prescribed vortioxetine, self-reported emotional blunting, depressive symptoms, cognitive dysfunction, functional impairment, and fatigue decreased over eight weeks. Anhedonia scores (SHAPS) decreased to non-significant levels, and clinician-rated Clinical Global Impression scores confirmed a significant reduction in illness severity.

## 1. Introduction

Major Depressive Disorder (MDD)* is a prevalent mental illness affecting an estimated 3.8% of the global population, equivalent to over 280 million individuals worldwide [1]. MDD remains a leading cause of disability. The World Health Organization (WHO) projects that by 2030 MDD will rank as the second leading cause of disability-adjusted life years (DALYs) globally [2]. A population-based study in Hong Kong has documented substantial increases in antidepressant prescribing over time, with selective serotonin reuptake inhibitors (SSRIs) being the most commonly used class, and a growing use of “other” antidepressants. These trends suggest rising antidepressant exposure, and thus antidepressant-associated emotional blunting—in local clinical practice, highlighting the relevance of evidence on switching to vortioxetine [3,4].

Emotional blunting involves numbing or flattening of both positive and negative emotions. It manifests as emotional indifference, reduced sensitivity, and diminished responsiveness, often reducing patients’ emotional range—including feelings of love, affection, fear, and anger, and profoundly impairing daily functioning [5,6]. Affected patients frequently experience adverse impacts on interpersonal relationships at home and in the workplace, reporting detachment from family, parenting challenges, and decreased sociability. Despite its clinical importance, recent surveys indicate that healthcare professionals often underestimate the impact of emotional blunting on patients’ daily lives [7].

The etiology of emotional blunting remains unclear; there is debate over whether it is a core MDD symptom or a side effect of antidepressants [8]. Some researchers posit that emotional blunting represents an underrecognized symptom of depression, often insufficiently captured by conventional assessment scales and inadequately addressed by selective serotonin reuptake inhibitors (SSRIs). Research has demonstrated associations between the severity of emotional blunting and the severity of depression in patients undergoing antidepressant therapy [5]. Notably, an online survey found no significant correlation between depression severity and emotional blunting prior to antidepressant treatment; however, 60% of patients reported emotional blunting following initiation of antidepressant therapy [8]. Furthermore, it is estimated that approximately 40–60% of MDD patients treated with SSRIs or serotonin–norepinephrine reuptake inhibitors (SNRIs) experience varying degrees of emotional blunting. In another study, it was estimated that approximately 40–60% of patients suffering from MDD who were treated with either SSRIs or SNRIs experienced some degree of emotional blunting [9].

Emotional blunting may be a consequence of reduced dopamine or glutamatergic activity [10]. Selective serotonin reuptake inhibitors (SSRIs) have a high serotonin transporter occupancy which may lead to negative downstream effects on dopamine and associated emotional blunting [11]. Vortioxetine, approved by the European Medicines Agency (EMA) in 2013 for MDD at 5–20 mg/day, belongs to the bis-aryl-sulfanyl amines class. It belongs to a new chemical class of psychotropics, the bis-aryl-sulfanyl amines. The mechanism of action is claimed to be related to its multimodal activity, which involves a combination of direct modulation of receptor activity and inhibition of the serotonin transporter. Serotonin (5-HT) receptors modulate mesocorticolimbic dopamine (DA) neuron firing and DA release in prefrontal and limbic targets, and this joint regulation of cortical–limbic circuits underlies individual differences in emotional reactivity. The effects of the 5-HT receptor on DA regulation are usually indirect and mediated by complex neuronal circuitry [12]. Vortioxetine was proven to be a safe and effective medication in many clinical trials and in meta-analyses, both short- and long-term. Vortioxetine may contribute toward alleviating emotional blunting via its multimodal mechanism of action [13], as it does not have a downstream negative effect on dopamine but rather a positive effect [14]. In a recent open-label study, vortioxetine was shown to improve emotional blunting effectively in MDD patients with partial response to SSRIs/serotonin–noradrenaline reuptake inhibitors (SNRIs) [15].

Unlike emotional blunting, anhedonia specifically involves reduced pleasure from rewarding stimuli, tied to mesolimbic dopamine dysfunction. In DSM-5 Criterion A, anhedonia is a core MDD symptom (markedly diminished interest or pleasure), while emotional blunting is not explicitly listed but relates to affective flattening [16,17]. It may arise from impairments in various facets of reward processing, including desire for reward, anticipation of reward, effort to attain reward, consummatory pleasure, and cognitive aspects of learning stimulus–reward associations—key characteristics of Major Depressive Disorder (MDD) [18]. This study assesses both emotional blunting and anhedonia, using the Snaith–Hamilton Pleasure Scale (SHAPS) for anhedonia.

Hong Kong, a high-prevalence metropolitan area for depression (2.9% for depressive episodes; 6.9% for mixed anxiety–depression), lacks data on emotional blunting severity in MDD patients [19]. Survey and observational data show that emotional blunting is one of the most prominent adverse effects prompting patients to discontinue antidepressant treatment, with more than one third of patients in large samples reporting stopping medication for this reason. Since blunting undermines motivation, decision-making, and engagement with treatment, it directly threatens adherence and can prematurely end otherwise effective pharmacotherapy [9,20]. Currently, the evidence is unclear about the clinical effectiveness of vortioxetine toward alleviating emotional blunting in MDD patients. Hence, this study aims to describe changes in emotional blunting and related clinical outcomes over eight weeks in a naturalistic cohort of patients with MDD who were prescribed vortioxetine in routine practice and to generate hypotheses for future controlled trials.

## 2. Materials and Methods

### 2.1. Study Design and Participants

This was a naturalistic longitudinal observational study conducted in Hong Kong. Patients with emotional blunting problems prescribed to start vortioxetine were recruited by psychiatrists. Eligible patients were aged 18 or above, were clinically diagnosed with MDD, and signed their written informed consent. The diagnosis criteria were based on DSM-5. Participants were approached after their psychiatrist’s decision to initiate vortioxetine; no minimum score on the Oxford Depression Questionnaire (ODQ) was required for enrollment. Patients with comorbidities involving other mental disorders were not excluded. We excluded individuals who had used vortioxetine within the preceding six months or were enrolled in another clinical trial.

Our study was approved by the Institutional Review Board of the University of Hong Kong/Hospital Authority Hong Kong West Cluster (HKU/HA HKW IRB UW 22-288).

### 2.2. Study Procedure

This study consisted of 1 brief intake interview and 4 self-report questionnaires. The ODQ, PHQ-9, Perceived Deficit Questionnaire Depression (PDQ-D), Sheehan Disability Scale (SDS), Multidimensional Fatigue Inventory (MFI), and Snaith–Hamilton Pleasure Scale (SHAPS) scores were assessed at baseline (week 0) and also at weeks 1, 4, and 8. Demographics such as age, gender, education level, monthly income, duration of current depressive episode, history of depressive disorder, past psychiatric history, past medical history, and past medication history were recorded. The questionnaires were completed online via Qualtrics, or over the phone administered by researchers. This study lasted for approximately 2 months. All online survey information was kept confidential, and data security was ensured to protect participating patients.

### 2.3. Study Endpoints

The primary endpoint was change in emotional blunting from baseline to week 8 (assessed by ODQ).

The secondary endpoints were changes from baseline to week 8 for the following: depression severity (PHQ-9); cognitive symptoms (PDQ-D); overall functioning (SDS); fatigue (MFI); anhedonia (SHAPS); Clinical Global Impression (CGI).

### 2.4. Assessment Tools and Outcome Measures

The ODQ score was used as the primary outcome [17]. This scale is a patient-reported rating scale, consisting of 26 items to assess five dimensions of emotional blunting: not caring (NC), emotional detachment (ED), positive reduction (PR), general reduction (GR), and antidepressant as cause (AC). Each item is rated on a 5-point Likert scale ranging from 1 (disagree) to 5 (agree). All item scores are summed for each dimension and a total score, ranging from 26 to 130, with higher scores meaning higher levels of emotional blunting. ODQ total score ≥ 50 represents substantial emotional blunting. 

The PHQ-9, PDQ-D, SDS, MFI, SHAPS, and CGI scores were used as secondary outcomes. These scales were all patient-rated and were used to assess the effectiveness of vortioxetine in treating depressive and cognitive symptoms.

The PHQ-9 is a 9-item scale rated by patients used to assess how much patients have been bothered by their symptoms over the past 2 weeks [21]. Each item is rated on a 4-point Likert scale ranging from 0 (not all all) to 3 (nearly every day). All item scores are summed for a total score, ranging from 0 to 27, with higher scores indicating greater depression severity: none (0–4), mild (5–9), moderate (10–14), moderately severe (15–19), and severe (20–27) depression.

The PDQ-D is a 20-item questionnaire rated by patients to assess cognitive dysfunction in the past 7 days [22]. It comprises 4 sub-scales identifying domains commonly impaired in depression: attention/concentration; prospective memory, planning/organization, and retrospective memory. Each item is rated on a 5-point scale ranging from 0 (never in the past 7 days) to 4 (very often—more than once a day). All item scores are summed for all sub-scales and a total score, ranging from 0 to 80, with higher scores indicating greater perceived cognitive impairment.

The SDS is a 3-item scale rated by patients to assess functional impairment in terms of work/school, social life, and family life/home responsibilities in the last week [23]. Each item is rated using a 10-point visual analog scale. All item scores are summed for a total score, ranging from 0 to 30, with higher scores indicating greater functional impairment.

The MFI is a 20-item scale rated by patients to assess five dimensions of fatigue: general fatigue, physical fatigue, reduced motivation, reduced activity, and mental fatigue [24]. Each item is rated on a 5-point Likert scale ranging from 1 (yes, that is true) to 5 (no, that is not true). All item scores are summed for each dimension and a total score, ranging from 20 to 100, with higher scores indicating higher levels of fatigue.

The SHAPS is a 14-item scale rated by patients to assess an individual’s pleasure experience in the “last few days” [25]. Each item is rated as a dichotomous score of 0 (agree) or 1 (disagree). All item scores are summed for a total score, ranging from 0 to 14, with higher scores indicating higher levels of anhedonia. SHAPS total score > 3 represents clinically significant anhedonia.

The Clinical Global Impression (CGI) rating scales are measures of symptom severity, treatment response, and treatment efficacy. The severity scale is rated on a 7-point scale ranging from 1 (normal, not at all ill) to 7 (among the most extremely ill patients) [26]. The improvement scale is also a 7-point scale requiring the clinician to assess how much the patient’s illness has improved relative to baseline. In addition, the efficacy index is a 4 × 4 rating scale that describes the degrees of therapeutic effect and side effects, the number representing where the two items intersect.

### 2.5. Statistical Analysis Plan

The prevalence of emotional blunting was estimated by the proportion of ODQ results over the cut-off score ≥ 50 and stratified by PHQ-9 level. The effectiveness of vortioxetine in treating emotional blunting (ODQ), depressive symptoms (PHQ-9), cognitive dysfunction (PDQ-D), functional impairment (SDS), fatigue (MFI), and pleasure experience (SHAPS) across the four timepoints was assessed using repeated-measures ANOVA. Post hoc analyses were performed for significant outcomes to identify significant comparisons. Paired *t*-test analysis was conducted for the change in CGI-S between baseline and 8-week follow-up. Correlation analyses of ODQ and PHQ-9 across all timepoints were performed to demonstrate the relationship between emotional blunting and depressive symptoms. Post hoc power analyses confirmed sample size sufficiency, with all statistical assumptions assessed and appropriate corrections applied. Statistical significance was set at *p* < 0.05. All statistical analyses were carried out with IBM SPSS Statistics for Mac, version 28.0.

## 3. Results

### 3.1. Descriptive Statistics

This research was based on a sample size of 87 respondents. Table 1 and Table 2 provide the sociodemographic and clinical profiles of the respondents.

### 3.2. Primary Outcome (Emotional Blunting)

Repeated-measures ANOVA was conducted to investigate the effects of vortioxetine on emotional blunting across four timepoints. Means and standard deviations are presented in Table 3, below.

**Figure 1:**
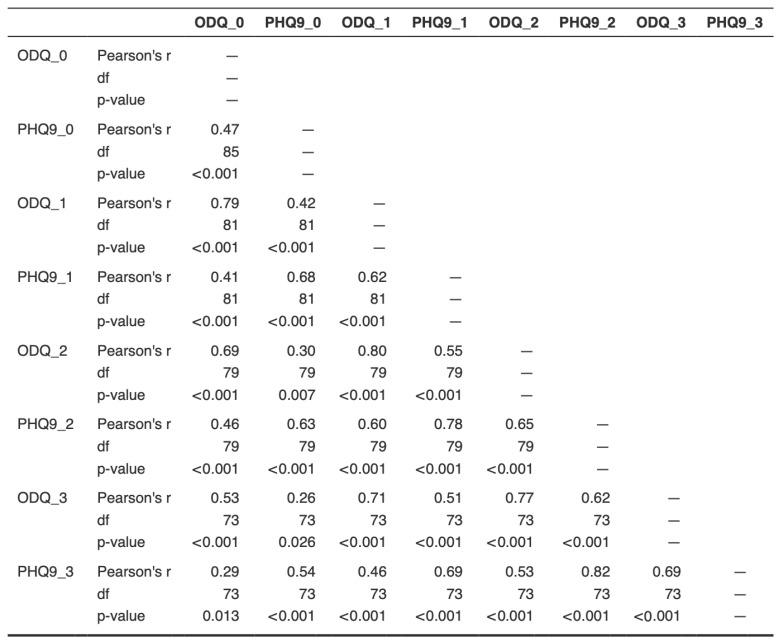
Correlation matrix of ODQ and PHQ-9 across 4 timepoints.

The prevalence of emotional blunting, estimated by the proportion of ODQ results over the cut-off score ≥ 50, was 91.9% at baseline, decreasing to 85.5% at week 1, 77.7% at week 4, and 73.3% at week 8. ODQ scores were significantly and positively associated with PHQ-9 at baseline (*r*s = 0.47, *p* < 0.001), 1-week follow-up (*r*s = 0.62, *p* < 0.001), 4-week follow-up (*r*s = 0.65, *p* < 0.001) and 8-week follow-up (*r*s = 0.69, *p* < 0.001) (Please refer to Figure 1).

**Figure 2:**
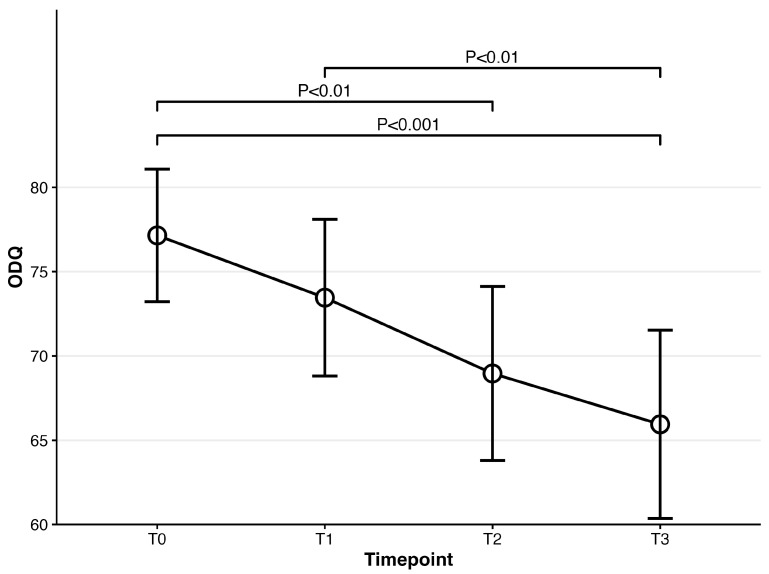
Mean scores for ODQ over time for patients prescribed vortioxetine.

The repeated-measures ANOVA on ODQ showed that patients who were prescribed vortioxetine showed a significant decrease across timepoints (F(3,222) = 10.56, *p* < 0.001). Post hoc analyses demonstrated that ODQ at baseline (mean: 77.15) was significantly different from that at 4-week follow-up (mean = 68.96, t(74) = 3.70, *p* = 0.002) and 8-week follow-up (mean = 65.95, t(74) = 4.19, *p* < 0.001). A significant difference between the 1-week and 8-week follow-up was also observed (1-week vs. 8-week: t(74) = 3.30, *p* = 0.008). No significant differences were observed in other pairs (Please refer to Figure 2). The ODQ remission rate, defined as a score < 50, at 8-week follow-up was 27% in the patients who were prescribed vortioxetine.

### 3.3. Secondary Outcome

Multiple repeated-measures ANOVAs were conducted to examine the effects of vortioxetine on depressive symptoms, cognitive dysfunction, functional impairment, fatigue, and increased pleasure experience across four timepoints. Means and standard deviations are presented in Table 4.

**Figure 3:**
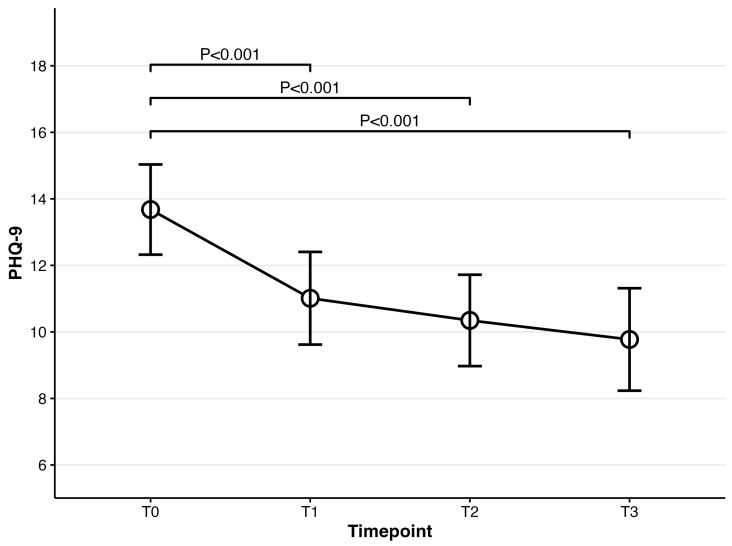
Mean scores for PHQ-9 over time for patients prescribed vortioxetine.

For depressive symptoms, the repeated-measures ANOVA on PHQ-9 showed that patients prescribed vortioxetine exhibited a significant decrease over time (*F*(3,222) = 17.20, *p* < 0.001). Compared to PHQ-9 at baseline (mean = 13.68), all timepoints showed a significant decline in post hoc analyses (1-week: mean = 11.01, t(74) = 6.70, *p* < 0.001; 4-week: mean = 10.35, t(74) = 5.23, *p* < 0.001; 8-week: mean = 9.77, t(74) = 5.29, *p* < 0.001). The remission rate of PHQ-9, defined as <5, was 24% at 8-week follow-up (Please refer to Figure 3).

**Figure 4:**
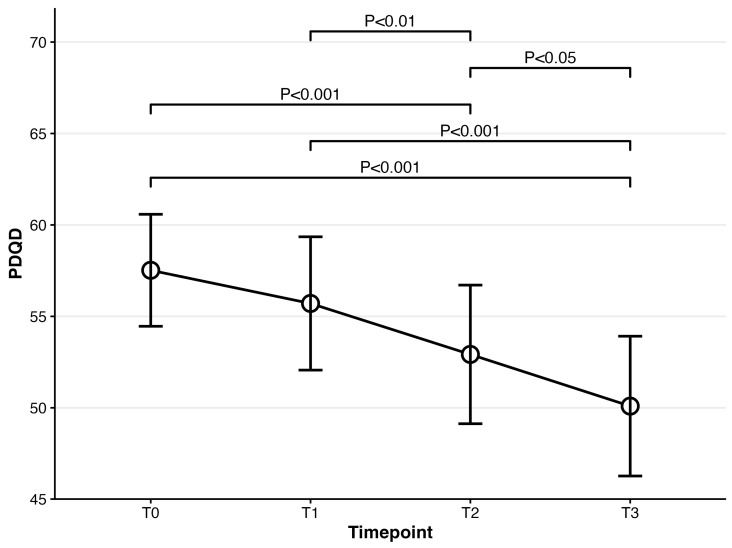
Mean scores for PDQ-D over time for patients prescribed vortioxetine.

**Figure 5:**
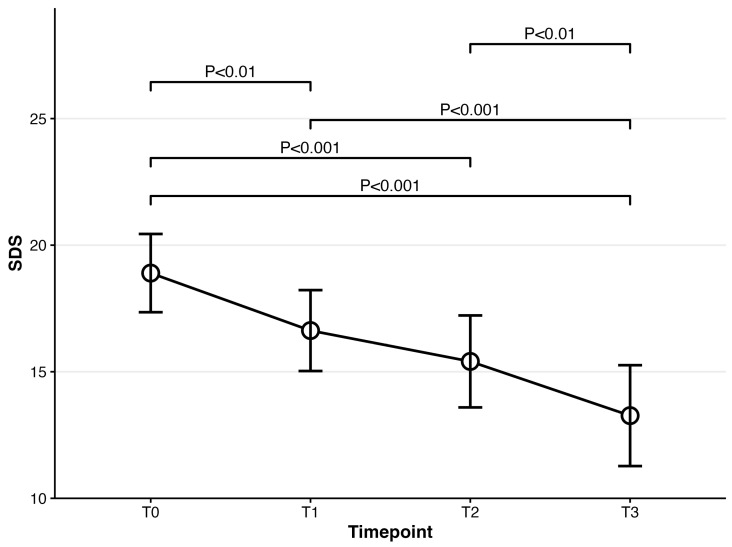
Mean scores for SDS over time for patients prescribed vortioxetine.

**Figure 6:**
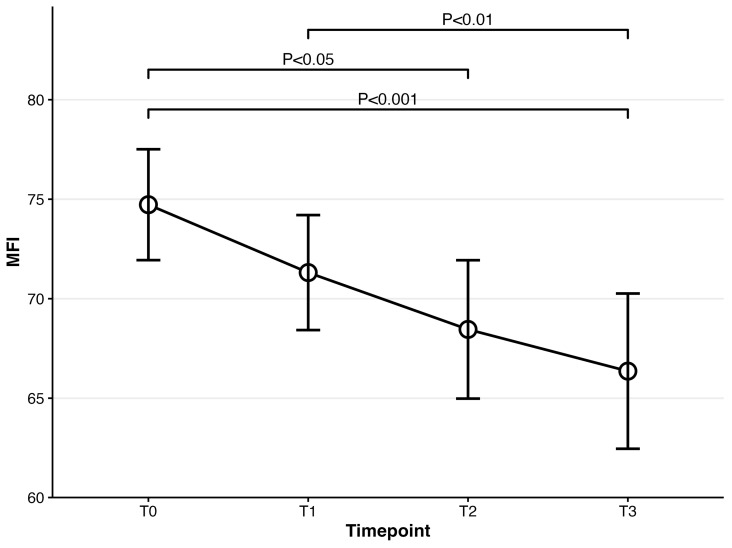
Mean scores for MFI over time for patients prescribed vortioxetine.

**Figure 7:**
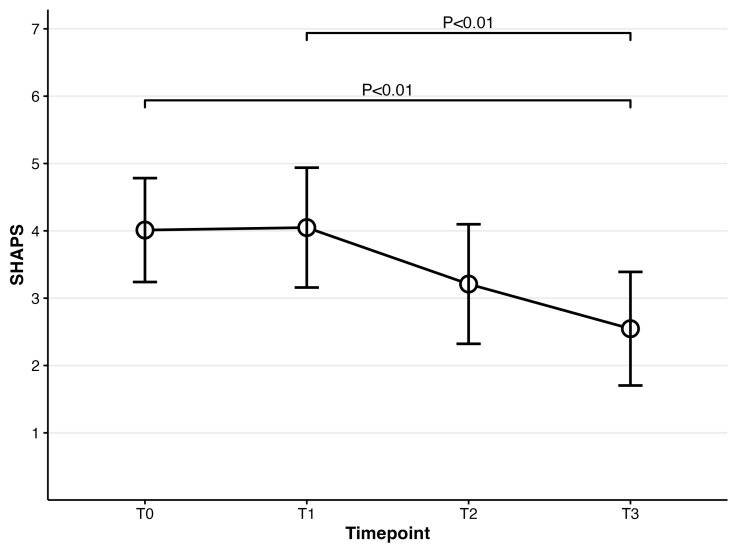
Mean scores for SHAPS over time for patients prescribed vortioxetine.

For cognitive dysfunction (PDQ-D), repeated-measures ANOVA was also significant at *F*(3,222) = 17.13, *p* < 0.001. Compared to PDQ-D at baseline, PDQ-D results at 4 weeks and 8 weeks were significantly lower (baseline: mean = 58.62; 4-week: mean = 53.01, t(74) = 3.91, *p* = 0.001; 8-week: mean = 50.09, t(74) = 5.32, *p* < 0.001). PDQ-D results at week 4 and week 8 were also significantly decreased compared to week 1 (1-week: mean = 56.02; 4-week: t(74) = 3.29, *p* = 0.009; 8-week: t(74) = 4.79, *p* < 0.001). A significant difference between week 4 and week 8 was also observed (t(74) = 2.83, *p* = 0.036) (Please refer to Figure 4). Regarding the severity of participants’ functional impairment (SDS), it was significant at *F*(3,222) = 19.60, *p* < 0.001. Post hoc analyses showed that all timepoints were significantly different from SDS at baseline (baseline: mean = 20.00; 1-week: mean = 16.63, t(74) = 3.58, *p* = 0.004; 4-week: mean = 15.41, t(74)= 4.21, *p* < 0.001; 8-week: mean = 13.27, t(74) = 6.29, *p* < 0.001). Significant differences were also observed in two pairs, including the pair of 1 week and 4 weeks and that of 4 weeks and 8 weeks (1-week vs. 4-week t(74) = 4.25, *p ≤* 0.001; 4-week vs. 8-week t(74) = 3.60, *p* = 0.003) (Please refer to Figure 5). Participants’ fatigue measured by MFI was significant at *F*(3,222) = 10.69, *p* < 0.001. Compared to MFI at baseline, MFI at week 4 and week 8 significantly decreased (baseline: mean = 74.72; 4-week: mean = 71.31, t(74) = 3.20, *p* = 0.012; 8-week: mean = 66.36, t(74) = 4.48, *p* < 0.001). MFI at week 1 was also significantly different from that at week 8 (t(74) = 3.51, *p* = 0.005) (Please refer to Figure 6). Lastly, the SHAPS score assessing patients’ pleasure experience was significant at *F*(3,222) = 8.02, *p* < 0.001. Significant differences were observed between baseline and week 8 and between week 1 and week 8 (baseline vs. 8-week: t(74) = 3.56, *p* = 0.004; 1-week vs. 8-week t(74) = 3.83, *p* = 0.002) (Please refer to Figure 7).

### 3.4. Effectiveness of Vortioxetine Based on Clinician’s Ratings

The CGI was developed as a brief, standalone tool for evaluating a patient’s global functioning before and after taking certain medications. The means and standard deviations for the effectiveness of vortioxetine are presented in Table 5, below.

In terms of the CGI, the mean severity score before taking vortioxetine was 4.90 (*SD* = 0.111), while the mean score after taking vortioxetine was 2.25 (*SD* = 0.144). A paired-samples *t*-test was conducted to determine the effect of vortioxetine on the severity of illness. The results indicated a significant difference between severity scores before taking vortioxetine and after taking vortioxetine [*t*(59) = 14.8, *p ≤* 0.001]. Meanwhile, in terms of participants’ improvement after taking vortioxetine, the mean score was 1.97 (*SD* = 0.116). Regarding the efficacy index, the mean score for the degree of therapeutic effect intersecting with side effects was 4.32 (*SD* = 0.491).

### 3.5. Post Hoc Power Analysis

Post hoc power analysis for the repeated-measures ANOVA was conducted using the observed effect size for ODQ (partial η^2^ = 0.12). The achieved power was greater than 0.999 at α = 0.05, suggesting that the study size was sufficiently powered to detect the observed effect size.

## 4. Discussion

In this study, patients with Major Depressive Disorder (MDD) treated with vortioxetine at doses ranging from 5 to 20 mg were associated with improvements in emotional blunting, depressive symptoms, cognitive dysfunction, functional impairment, and fatigue after eight weeks of treatment, though causal effects cannot be inferred. By study completion, 27% of patients no longer reported emotional blunting, compared with 92% at baseline. Notably, improvements in emotional blunting were evident as early as one week after treatment initiation.

Hong Kong Chinese individuals are often described as bicultural, influenced by both British and broader Western norms alongside traditional Chinese values. This bicultural context may shape norms regarding emotional restraint, acceptable expressions of positive affect, and willingness to acknowledge emotional problems—factors that affect whether reduced emotional expression is perceived as pathological “blunting” or as culturally appropriate control [27].

Emotional blunting has been hypothesized to involve serotonergic effects in the frontal lobes and serotonergic modulation of midbrain dopaminergic pathways projecting to the prefrontal cortex. Serotonergic modulation of striatal and prefrontal circuits shapes reward valuation and motivational drive, offering a mechanistic bridge to phenomena such as emotional numbing and reward suppression. Increases or decreases in serotonin signaling are proposed to rebalance circuit activity but can also shift sensitivity away from both aversive and rewarding outcomes, potentially contributing to blunted emotional experience in some treatment contexts [28]. Selective serotonin reuptake inhibitors (SSRIs) broadly enhance serotonergic transmission, activating gamma-aminobutyric acid (GABA) interneurons, which in turn inhibit dopaminergic inputs. Vortioxetine exhibits a distinct pharmacological profile characterized by 5-hydroxytryptamine (5-HT)1A agonism and 5-HT3, 5-HT1D, and 5-HT7 antagonism, as well as partial 5-HT1B agonism [29]. The blockade of 5-HT3 receptors on GABAergic interneurons may lead to increased levels of serotonin, dopamine, norepinephrine, acetylcholine, and histamine. This multimodal mechanism likely underpins vortioxetine’s ability to alleviate emotional blunting [12]. A prior study on the anxiety circuit emphasized that pathological states are not driven solely by limbic hyper-reactivity but also by maladaptive prefrontal control, with medial and dorsolateral prefrontal regions exerting excessive or misdirected influence over subcortical affect generators. When serotonergic modulation of these prefrontal hubs is abnormal, top-down control may become overgeneralized, suppressing both negative and positive emotional responses and thereby contributing to the subjective sense of “feeling nothing” that characterizes emotional blunting [28,30]. At the same time, disrupted 5-HT regulation of ventral striatal and orbitofrontal circuits involved in reward prediction and valuation can blunt reward responsivity and hedonic tone. This provides a plausible neurobiological bridge between serotonergic antidepressant exposure, reduced striatal reactivity to rewarding stimuli, and clinical reports of diminished motivation, interest, and pleasure despite partial mood improvement [31].

In this naturalistic cohort, there were significant positive correlations between ODQ and PHQ-9 at all timepoints. The moderate-to-high correlations between ODQ and PHQ-9 suggest considerable overlap between emotional blunting and depressive symptom severity at the level of self-reporting. The emotional blunting assessed by the ODQ cannot be cleanly separated into treatment-emergent and disorder-intrinsic components. Most participants had prior antidepressant exposure, and many reported blunting as a salient treatment-related complaint, yet emotional numbing is also recognized as part of the broader affective flattening seen in depression. Our findings should therefore be interpreted as reflecting the overall burden of emotional blunting in treated MDD, rather than a pure marker of drug-induced change or disease severity alone. However, emotional blunting did not simply mirror depressive severity. For example, although mean PHQ-9 scores decreased significantly over time, 73.3% of patients still met the ODQ clinical cut-off for substantial blunting at week 8, whereas PHQ-9 remission (score < 5) was achieved in 24%. This divergence between depressive remission rates and persistence of blunting supports the view that emotional blunting represents a related but only partially overlapping construct. Similarly, clinically significant anhedonia (SHAPS ≥ 3) declined to non-significant levels by week 8, whereas emotional blunting remained common, further indicating non-redundancy of ODQ relative to both mood and hedonic measures.

The prior literature supports these findings. A study involving 669 patients under depression treatment and 150 recovered controls reported that nearly half of antidepressant users experienced emotional blunting—a phenomenon common across monoaminergic antidepressants. Greater blunting severity correlated with poorer remission quality [5]. Clinically, reducing the dosage of an antidepressant or switching to a medication from a different drug class are common strategies to alleviate emotional blunting [9]. Substantial evidence suggested that emotional blunting is induced by antidepressant treatment and differs from the emotional states experienced during depressive episodes [32]. For instance, a qualitative study involving 220 participants currently or previously taking SSRIs found that most attributed their emotional numbing specifically to their medication [33].

Clinicians may underestimate the prevalence and functional impact of emotional blunting in depression. One study revealed that blunting impaired daily functioning more than mood, anxiety, cognitive, or physical symptoms, with 63–75% of acute-phase patients and 31–50% in remission reporting significant impairment across FAST domains such as autonomy, work, relationships, and leisure. Low well-being scores (WHO-5: 6.4 in acute phase, 9.8 in remission) highlighted a persistent burden even after mood improvement. Emotional blunting thus hinders full recovery, contributes to medication discontinuation, and warrants targeted recognition and management to restore function and quality of life [34].

In another study, almost three quarters of patients in the acute phase of depression and one quarter of those in remission reported severe emotional blunting. Approximately 56% of patients considered their emotional blunting to be caused by their depression, while 45% believed that their antidepressant medication was negatively affecting their emotions. As a result, over one third of patients were considering stopping or had stopped their antidepressant [35]. Vortioxetine has demonstrated superior improvement in emotional blunting compared with SSRIs, SNRIs, and agomelatine, significantly outperforming conventional antidepressants, with up to 70% of patients showing resolution after 8 weeks [36,37]. Across open-label and comparative trials, vortioxetine resolves blunting in 60–70% of cases within 8 weeks at higher doses (20 mg), surpassing the 30–50% partial response rates associated with SSRI/SNRI continuation. These effects likely reflect vortioxetine’s multimodal serotonergic actions, which enhance prefrontal and reward-circuit activity more effectively than traditional reuptake inhibitors or agomelatine’s melatonergic mechanisms [15].

This study showed that vortioxetine was associated with a reduction in emotional blunting while concurrently alleviating depressive symptoms. Consistent with findings from the existing literature, a study of patients with MDD who exhibited partial response to SSRI or SNRI monotherapy (at adequate doses for ≥6 weeks) and were subsequently switched to vortioxetine (10–20 mg/day) for 8 weeks reported that, by week 8, 50% of participants no longer experienced emotional blunting as assessed by standardized screening questions [15]. Similarly, in a randomized, placebo-controlled 6-week trial evaluating vortioxetine augmentation with the anti-inflammatory agent celecoxib versus placebo, vortioxetine demonstrated favorable effects on emotional blunting across both short- and long-term treatment courses. However, the beneficial impact was attenuated in celecoxib-augmented patients compared with those receiving placebo, potentially attributable to pharmacokinetic interactions [37]. These results align with the present study, providing further evidence for vortioxetine’s efficacy in mitigating emotional blunting.

However, a recent systematic review and meta-analysis reveals that vortioxetine response rates were lower than active serotonin and norepinephrine reuptake inhibitor (SNRI) comparators for the 5 mg, 15 mg, and 20 mg doses. The most common adverse events were nausea and vomiting, which increased in frequency with higher doses. The study reveals that vortioxetine does not appear to be more effective and is potentially less effective than an SNRI, underscoring the need to temper broad claims of clinical superiority [34].

Concerning the strengths of this study, this study represents one of the first real-world studies exploring the change in emotional blunting, cognitive dysfunction, functional impairment, and fatigue with the use of vortioxetine. Additionally, the incorporation of clinician ratings enhances the study’s reliability and mitigates potential subject bias.

This study has several limitations. First, given the uncontrolled, observational nature of this study, the observed reductions in emotional blunting and other symptoms over eight weeks cannot be attributed solely to vortioxetine. Potential contributors include regression to the mean, natural symptom fluctuation, concurrent interventions, and expectancy effects. The present findings should therefore be interpreted as descriptive, hypothesis-generating real-world data rather than definitive evidence of treatment effectiveness. Second, although most participants had prior antidepressant exposure, this limits the ability to differentiate blunting caused by prior medication from that inherent to depression. The lack of washout control, dosage information, and baseline severity data further compromises internal validity. A comparison group receiving other SSRIs or SNRIs would have clarified vortioxetine’s specific effects. Third, the inclusion of all participants initiating vortioxetine, without stratification by baseline emotional blunting severity, may have introduced floor effects and compromised validity. Additionally, reliance on self-reported measures without external validation raises concerns about accuracy. Clinicians were also not routinely prompted to assess emotional side effects, reducing tracking consistency. The lack of biomarker correlation and the short duration of this study are other noteworthy limitations. Finally, the substantial correlation between ODQ and PHQ-9 scores across timepoints indicated notable construct overlap between emotional blunting and depressive severity in our sample. Because all measures were self-report scales administered contemporaneously, shared method variance likely inflated these associations and limited inferences about the independence of emotional blunting as measured here.

## 5. Conclusions

In this naturalistic sample of Hong Kong patients with MDD who were prescribed vortioxetine, self-reported emotional blunting and other clinical outcomes improved over eight weeks. These observational findings generate hypotheses that vortioxetine may be a useful option for patients troubled by emotional blunting, but randomized controlled trials and comparative effectiveness studies are required to determine causal efficacy and to quantify benefits relative to alternative treatments.

## Figures and Tables

**Table 1:** Sociodemographic characteristics of participants at baseline. N = 87.

Baseline Characteristic	Level	Full Sample	
		*n*	%
Gender	Female	48	55.2
	Male	39	44.8
Marital status	Single	52	59.8
	Married	26	29.9
	Divorced	5	5.7
	Widowed	3	3.4
	Other	1	1.1
Highest educational level	No education	1	1.1
	Primary	4	4.6
	Secondary	23	26.4
	College	13	14.9
	University	25	28.7
	Master’s	21	24.1
Employment	Student	11	12.6
	Housewife	39	44.8
	Full-time	7	8.0
	Part-time	8	9.2
	Unemployed	19	21.8
	Retired	3	3.4
Monthly income	Under 5999	28	32.2
	6000–14,999	8	9.1
	15,000–29,999	21	24.1
	30,000 or above	30	34.4
Able to cover living expense	Yes	64	73.6
	No	23	26.4

**Table 2:** Characteristics of participants’ medical histories at baseline. N = 87.

Baseline Characteristic	Level	Full Sample	
		*n*	%
Number of years with mood disorder symptoms	1 to 3	44	50.6
	4 to 6	9	10.3
	7 to 9	5	5.7
	10 or above	29	33.3
Year since first diagnosed with mood disorder	1 to 3	56	64.4
	4 to 6	7	8.0
	7 to 9	7	8.0
	10 or above	17	19.5
Past admission to psychiatric facility	Yes	8	9.2
	No	79	90.8
History of suicide attempt	Yes	8	9.2
	No	79	90.8
Family history of mental illness	Yes	24	27.6
	No	63	76.4
Dosage	5 mg	42	48.3
	10 mg	32	36.8
	15 mg	4	4.6
	20 mg	6	6.9
	Others	3	3.4
Previously taken antidepressants	SSRI	76	87.4
	SNRI	5	5.7
	Combination of SSRI and SNRI	5	5.7
	Missing	1	1.1
Psychiatric comorbidity	Yes	51	58.6
	No	36	41.3

**Table 3:** Means, standard deviations, and repeated-measures ANOVA for emotional blunting.

Measure	Baseline (N = 87)		1-Week (N = 83)		4-Week (N = 81)		8-Week (N = 75)		*F (3,222)*	*p Value*	Partial η^2^
	***M***	***SD***	***M***	***SD***	***M***	***SD***	***M***	***SD***			
*ODQ*	77.15	18.43	73.46	21.29	68.96	23.33	65.95	24.29	*10.56*	<0.001 ***	0.12

*Note:* ODQ = Oxford Depression Questionnaire; * *p* < 0.05, ** *p* < 0.01, *** *p* < 0.001.

**Table 4:** Means, standard deviations, and repeated-measures ANOVA for secondary outcomes.

Measure	Baseline (N = 87)		1-Week (N = 83)		4-Week (N = 81)		8-Week (N = 75)		*F (3,222)*	*p Value*	Partial η^2^
	*M*	*SD*	*M*	*SD*	*M*	*SD*	*M*	*SD*			
*PHQ-9* ^	13.68	6.36	11.01	6.38	10.35	6.21	9.77	6.70	17.20	<0.001 ***	0.19
*PDQ-D* ^	58.62	13.45	56.02	15.91	53.01	16.63	50.09	16.61	17.13	<0.001 ***	0.19
*SDS ^*	20.00	7.25	16.63	7.32	15.41	8.21	13.27	8.66	19.60	<0.001 ***	0.21
*MFI* ^	74.72	13.07	71.31	13.23	68.46	15.73	66.36	16.95	10.69	<0.001 ***	0.13
*SHAPS*	4.01	3.62	4.05	4.08	3.21	4.02	2.55	3.67	8.02	<0.001 ***	0.10

*Note:* PHQ-9 = Patient Health Questionnaire-9, PDQ-D = Perceived Deficits Questionnaire—Depression, SDS = Sheehan Disability Scale, MFI = Multidimensional Fatigue Inventory, SHAPS = Snaith–Hamilton Pleasure Scale; * *p* < 0.05, ** *p* < 0.01, *** *p* < 0.001. ^ Huynh–Feldt correction was adopted due to violation of sphericity.

**Table 5:** Means, standard deviations, and paired *t*-test results for Clinical Global Impression.

Measure	Baseline (N = 87)		8-Week (N = 80)		*t*	*p Value*
	*M*	*SD*	*M*	*SD*	14.8	<0.001 ***
*CGI-MI*	4.90	0.858	2.25	1.11		
*CGI-CHANGE*			1.97	0.116		
*CGI-EFFECT*			4.32	0.491		

Note: CGI = Clinical Global Impression, MI = severity scale, CHANGE = improvement scale, EFFECT = efficacy index, * *p* < 0.05 and ** *p* < 0.01, *** *p* < 0.001.

## Data Availability

The original contributions presented in this study are included in the article. Further inquiries can be directed to the corresponding author.

## Abbreviations

The following abbreviations are used in this manuscript:

MDD	Major Depressive Disorder
SSRI	Selective serotonin reuptake inhibitor
EMA	European Medicines Agency
ODQ	Oxford Depression Questionnaire
PHQ-9	Patient Health Questionnaire-9
PDQ-D	Perceived Deficits Questionnaire—Depression
SDS	Sheehan Disability Scale
MFI	Multidimensional Fatigue Inventory
SHAPS	Snaith–Hamilton Pleasure Scale
CGI	Clinical Global Impression; MI = severity scale, Change = improvement scale, Effect = efficacy index
